# Mobile-Based Interventions for Dietary Behavior Change and Health Outcomes: Scoping Review

**DOI:** 10.2196/11312

**Published:** 2019-01-21

**Authors:** Atreyi Kankanhalli, Jieun Shin, Hyelim Oh

**Affiliations:** 1 Department of Information Systems and Analytics National University of Singapore Singapore Singapore

**Keywords:** mHealth, mobile-based intervention, dietary behavior, food intake, behavior change, health outcomes

## Abstract

**Background:**

Mobile apps are being widely used for delivering health interventions, with their ubiquitous access and sensing capabilities. One such use is the delivery of interventions for healthy eating behavior.

**Objective:**

The aim of this study was to provide a comprehensive view of the literature on the use of mobile interventions for eating behavior change. We synthesized the studies with such interventions and mapped out their input methods, interventions, and outcomes.

**Methods:**

We conducted a scoping literature search in PubMed/MEDLINE, Association for Computing Machinery Digital Library, and PsycINFO databases to identify relevant papers published between January 2013 and April 2018. We also hand-searched relevant themes of journals in the *Journal of Medical Internet Research* and registered protocols. Studies were included if they provided and assessed mobile-based interventions for dietary behavior changes and/or health outcomes.

**Results:**

The search resulted in 30 studies that we classified by 3 main aspects: input methods, mobile-based interventions, and dietary behavior changes and health outcomes. First, regarding input methods, 5 studies allowed photo/voice/video inputs of diet information, whereas text input methods were used in the remaining studies. Other than diet information, the content of the input data in the mobile apps included user’s demographics, medication, health behaviors, and goals. Second, we identified 6 categories of intervention contents, that is, self-monitoring, feedback, gamification, goal reviews, social support, and educational information. Although all 30 studies included self-monitoring as a key component of their intervention, personalized feedback was a component in 18 studies, gamification was used in 10 studies, goal reviews in 5 studies, social support in 3 studies, and educational information in 2 studies. Finally, we found that 13 studies directly examined the effects of interventions on health outcomes and 12 studies examined the effects on dietary behavior changes, whereas only 5 studies observed the effects both on dietary behavior changes and health outcomes. Regarding the type of studies, although two-thirds of the included studies conducted diverse forms of randomized control trials, the other 10 studies used field studies, surveys, protocols, qualitative interviews, propensity score matching method, and test and reference method.

**Conclusions:**

This scoping review identified and classified studies on mobile-based interventions for dietary behavior change as per the input methods, nature of intervention, and outcomes examined. Our findings indicated that dietary behavior changes, although playing a mediating role in improving health outcomes, have not been adequately examined in the literature. Dietary behavior change as a mechanism for the relationship between mobile-based intervention and health outcomes needs to be further investigated. Our review provides guidance for future research in this promising mobile health area.

## Introduction

### Technology and Healthy Eating Promotion

Changes in lifestyle have resulted in dietary problems, such as consumption of high-calorie and low-nutrient foods. Consumption of such foods is associated with obesity and chronic diseases, such as cardiovascular disease and diabetes [1-3]. The problem of widespread obesity is a serious public health concern for individuals, care providers, and policy makers [2]. To mitigate these issues, technological interventions are being developed to encourage people to consume a diversified, balanced, and healthy diet depending on individual needs (eg, age, gender, and lifestyle), cultural context, locally available foods, and dietary customs. According to the Food and Agriculture Organization, most people make their dietary choices for personal reasons, for example, based on time constraints and convenience, personal preferences, and everyday habits [4], rather than basing them on good nutrition and health. Although individuals often realize that their eating behaviors are not ideal for their health and endeavor to change them, failure to maintain a long-lasting, healthy lifestyle is very common [5]. In this regard, technological interventions have the potential to assist in encouraging users to consume a healthy diet.

### Mobile-Based Health Interventions

Particularly, mobile-based interventions have become a popular means for the promotion and continuity of self-management of users’ health [6]. The distinct features that facilitate the adoption of mobile health (mHealth) apps are the ubiquity and sensing capabilities of mobile devices. Compared with Web-based interventions, mobile apps enable users to log their food intake behaviors and other activities throughout the day. Moreover, the technical capabilities of mobile devices equipped with sensors and computing power have enabled mobile app designers to develop interactive interventions that facilitate monitoring and self-management of health behaviors, such as physical activity [7,8].

At the same time, health psychology literature identifies various behavior change techniques that can be used as interventions to promote healthy behavior [9,10]. The change techniques cited include providing general health information, instructions, prompt reviews of behavior goals, and self-monitoring of users’ behavior. Physical activity and eating behaviors are 2 of the most targeted health behaviors, because of their importance for health outcomes. In this regard, there is considerable literature reviewing the interventions for physical activity, for example, exercise and fitness [7,8].

With diverse behavior change techniques, mobile interventions on users’ diets can lead to weight loss, diabetes management, or health promotion, in general [11-14]. However, despite the need for a systematic investigation of such interventions for dietary behavior changes, there are limited related reviews [15,16]. This could be partly because of the erstwhile difficulties of measuring a person’s dietary behavior and then improving on it [15-17]. As one approach, Roy et al [18] assessed healthy eating behavior by measuring if patients’ diets are healthy through allocating scores for consuming a variety of foods or recommended foods and nutrients. Further, in prior research on mobile interventions for dietary behavior changes, healthy eating behavior is suggested as a mediator for health outcomes because it is a crucial step for attaining the outcomes [13,19-32]. Yet, some studies only examined the dietary behavior changes, for example, fruit and vegetable intake change, after mobile-based interventions [19-24,27-30]. We found only a few studies assessing both dietary behavior changes and health outcomes [13,25,26,31,32]. However, other researchers have directly studied the effects of mobile-based interventions on health outcomes (eg, weight loss) [11,12,14,33-42].

### Research Gap

To the best of our knowledge, previous literature reviews have not incorporated a comprehensive list of studies on mobile interventions for dietary behavior change. Bardus et al [43], DiFilippo et al [15], and Nour et al [16] are the closest to our scoping review, yet their focus differs from our review. Bardus et al [43] examined both the use of mobile phones and websites for weight management, and mainly focused on the comparison between these 2 technologies. DiFilippo et al [15] included only 4 studies in their review, which evaluated weight loss as the outcome of better nutrition, while excluding mobile interventions using text messaging or digital photography. Nour et al [16] focused on mobile interventions whose objective was limited to increasing vegetable intake in young adults. In contrast, we aimed to review the mobile interventions for healthy eating more broadly by synthesizing all studies that focus on mobile interventions for dietary behavior change and health outcomes.

### Objectives

The objectives of this scoping review were to identify and synthesize the existing literature on mobile-based interventions for dietary behavior changes and health outcomes. This enables us to better understand the mobile interventions that affect user’s eventual health outcomes, which can lead to more efficient and effective promotion of diet guidelines, and improved consumer health and lifestyle policies. Specifically, our review sought to identify and categorize 3 main aspects: (1) the diet input methods of these mobile apps; (2) the mobile-based interventions; and (3) the dietary behavior changes and health outcomes. In addition, we coded the study sample characteristics and methods. Our general research question that guided this scoping review is as follows: “How do mobile interventions influence dietary behavior changes and health outcomes?”

## Methods

### Scoping Review Methodology

Given the rapid evolution of mHealth apps, we chose a scoping review methodology to obtain an overview of the extant literature on mobile interventions for dietary behavior changes and health outcomes. A scoping review is a literature review technique that is useful to *map* relevant literature in a field of interest [44,45]. At a general level, a scoping review aims “to map rapidly the key concepts underpinning a research area and the main sources and types of evidence available, and can be undertaken as stand-alone projects in their own right, especially where an area is complex or has not been reviewed comprehensively before” [46]. Therefore, a scoping review addresses broader topics where many different study designs might be applicable [47]. On the contrary, a systematic review answers a well-defined question from studies with appropriate designs, which typically focus on randomized controlled trials (RCTs) or quality-assessed studies with a relatively narrow range to synthesize evidence from them [48].

As described earlier, the literature on dietary behavior changes through mobile interventions has yet to be comprehensively reviewed, thus motivating our scoping review. Furthermore, although 20 of the studies in our review [11-13,19-21, 23,24,28,30-37,40,42,49] conducted RCTs, they differed in research questions and objectives, and had diverse outcome variables. Thus, the effectiveness of different interventions in these studies becomes incomparable through a systematic review. In this sense, it was more meaningful to conduct a scoping review for mapping out diverse literature on mobile interventions for dietary behavior changes and health outcomes.

### Identifying Relevant Studies and Study Selection

#### Search Strategy

A scoping literature search was performed on the PubMed/MEDLINE, Association for Computing Machinery Digital Library, and PsycINFO databases. Additionally, we did hand-searches through all relevant themes of journals in *Journal of* *Medical Internet Research* (JMIR) and through the registered and published protocols in PROSPERO. The search was restricted to publications from January 2013 to April 2018. The reason that we chose to start the search from 2013 is that the rise in popularity of mobile apps began then. These databases were searched for relevant publications in fields of the title, abstract and keywords using the following search terms: “([food OR diet OR nutrition OR intake] and [mHealth OR mobile OR smartphone OR mobile application]).”

#### Eligibility and Exclusion Criteria

Our aim was to include papers that describe mobile-based interventions for dietary behavior changes and/or health outcomes. Studies were included if they (1) were an original paper published in peer-reviewed journals (except review papers); (2) included mobile-based interventions to influence users’ dietary behavior; and (3) reported dietary behavior changes or health outcomes from the mobile-based interventions. With respect to dietary behavior changes (eg, eating more vegetables and consuming food with fewer calories), biochemical outcomes (eg, blood glucose and urinary sodium changes), and health status changes (eg, weight loss), studies targeting multiple health behavior changes/outcomes (eg, changes in both dietary behavior and health status) were also included, as long as at least one change or outcome was related to diet.

Regarding our exclusion criteria, studies dealing with consumption of drugs, toxic substances, chemicals, or pharmaceutical elements were not included. Studies targeting only people with specific diseases or disorders, such as AIDS, cancer, or mental disorder, were also excluded. However, we included studies targeting people who are obese or diabetic as these diseases are directly related to dietary behavior and are more common in the general public. Furthermore, studies focusing on eating disorders such as anorexia and binge eating behavior were not included as the results would not be applicable to a large population. Similarly, studies targeting very specific groups of people, such as pregnant women, children, or athletes, were excluded as the necessary components of their diet, such as minerals (eg, zinc), are not applicable to the broader population. Additionally, studies about the design, development, usability, acceptability, or feasibility of mHealth apps are not within the scope of this review.

#### Study Selection

**Figure 1:**
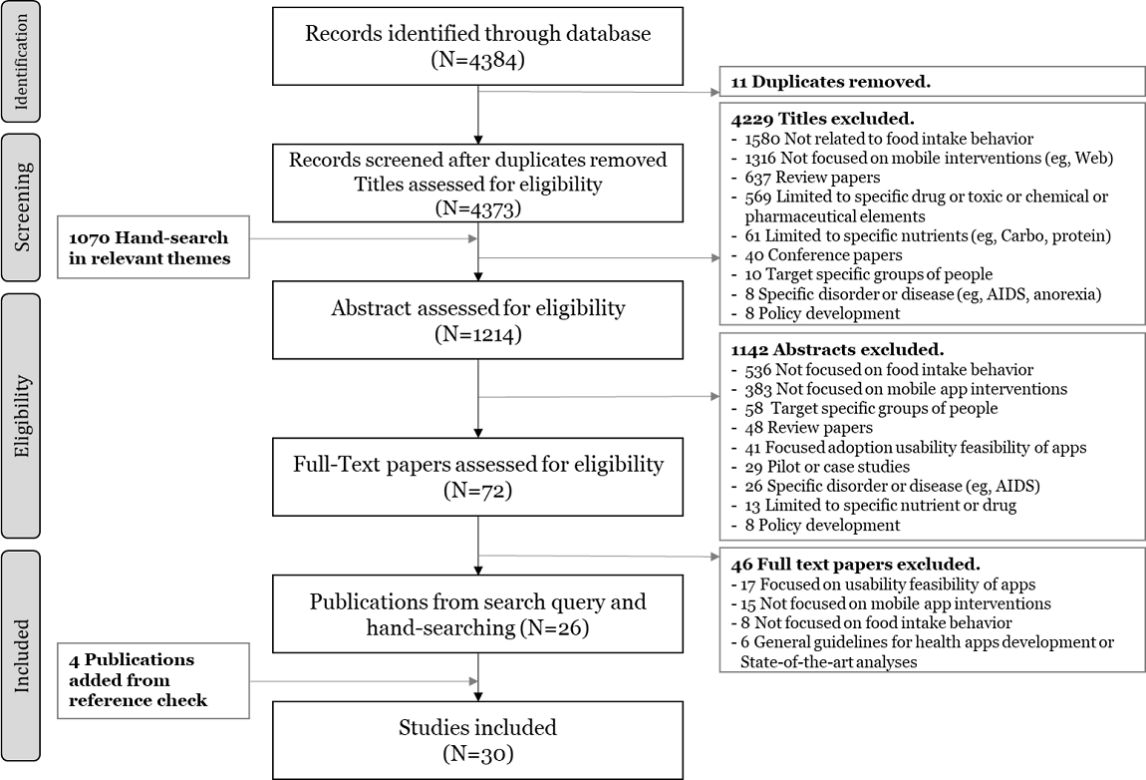
Flowchart of the selection process for the scoping review.

We downloaded the titles and abstracts of all screened studies and used EndNote X8 (Thomson Reuters) for citation management. Duplicates were removed, and the titles and abstracts were reviewed by grouping papers into 4 categories: (1) studies meeting our selection criteria; (2) studies requiring further examination; (3) excluded studies; and (4) other review papers. The selection of studies for our research was conducted and reported according to the guidelines for conducting scoping reviews [47]. In the searching stage, the reference list of all identified reports and papers was searched to include additional studies. Furthermore, 3 reviewers discussed the inclusion and exclusion criteria, and 2 reviewers independently reviewed abstracts for inclusion. Subsequently, papers that were determined to be potentially relevant to our review were downloaded in entirety and reviewed for eligibility. In sum, our search identified 5607 papers, of which 26 studies met the inclusion criteria. In addition, 4 studies were added from reference checks, as the scoping review methodology allows to refine the search strategy during the selection process [43,49,50]. As a result, 30 studies were selected for our final list. The complete selection process is illustrated in Figure 1.

#### Extraction and Charting of Results

Following study selection, data extraction was completed according to the standard practice for high-quality scoping reviews. A data-charting form was created by the research team to include study characteristics, mobile app input characteristics, mobile-based intervention characteristics, and outcomes of the study. The components of the mobile interventions were classified by a set of behavior change techniques that were seen to have an effect on health behavior [9,10,51]. These included self-monitoring, feedback, gamification, goal reviews, social support, and educational information (Textbox 1). All relevant data from the studies were coded using the data-charting form, and short summaries were obtained to provide an overview of the included studies presented in Multimedia Appendix 1.

**Components of the data-charting form.**

**Study characteristics:**

- Title
- Author (year)
- Participants
- Country of the study
- Study method(s)
- Duration of the study

**Mobile app input characteristics:**

- Modes of input data
  - Audio/video/photo recognition
  - Text
- Contents of input data
  - Demographic information
  - Medication
  - Health measures
  - Goals
  - Food intake (diet)

**Mobile intervention characteristics:**

- Modes of mobile interventions
  - In-app log
  - In-app feedback
  - Notification from mobile app
  - Other notification (short message service and email)
- Content of mobile interventions
  - Self-monitoring
  - Feedback
  - Gamification
  - Goal-setting and review
  - Social support
  - Educational information
- Related theories
  - Learning theory (LT)
  - Theory of planned behavior (TPB)
  - Social cognitive theory (SCT)
  - Self-regulation theory (SRT)
  - Theory of behavior changes (TBC)
  - Control theory (CT)
  - Self-determination theory (SDT)
  - Social network theory (SNT)
  - Processes-of-change theory (PCT)

**Outcomes of the study:**

- Dietary behavior changes (DBC)
- Biochemical outcomes (BO)
- Health status (HS)

## Results

### Overview

Our initial search identified 4384 papers from the databases. We also included 1201 hand-searched papers from relevant themes of journals in the *JMIR* journals, specifically from “mHealth for Wellness, Behavior Change and Prevention,” “Instruments and Questionnaires for Nutrition and Food Intake,” and “Mobile Health (mHealth).” Of these, we found that 131 studies had already been included through our previous searches. Thus, as per the procedure in Woodward et al’s study [52], we included only 1070 hand-searched papers in the selection process after removing duplicates. The details of the hand-search process and outcomes are illustrated in Figures A1-A3 in Multimedia Appendix 2. Our search in PROSPERO did not yield any additional protocols that met the inclusion criteria. Most of the papers (4229) were excluded during the title screening stage itself because there were a limited number of previous studies where a nutrition app had been used to change dietary behavior [15]. When reviewing the abstracts, we further excluded 1142 papers that did not focus on mobile interventions or dietary behavior or were limited to examining the adoption, usability, and feasibility of mobile apps. Of the remaining 72 papers assessed for full-text eligibility, 15 did not focus on mobile app interventions. Another 17 studies focused on the usability and feasibility of mobile apps, 8 did not focus on the food intake behavior, and 6 studies provided either general guidelines for app development or state-of-the-art analyses of mobile apps. Finally, a check of the reference lists of the included papers resulted in the addition of 4 more publications, bringing our total to 30 papers.

### Characteristics of Studies

More than a third of the studies (ie, 11) were conducted in the United States [11-13,26,27,30,32,35,41,49,50], followed by 7 studies in Australia [19,22,24,31,36-38]. In addition, 2 studies each were conducted in China [14,39], Canada [21,25], and Spain [28,40]. Furthermore, 1 study each was conducted in Austria [33], Finland [23], Japan [42], the Netherlands [20], Portugal [29], and Singapore [34]. In terms of the methods of the included studies, 20 studies [11-13,19-21,23,24,28, 30-37,41,42,49] conducted RCTs and 3 conducted field studies [22,27,50]. Among the field studies, Wharton et al [50] conducted a field study that randomized 3 groups, but did not have a control group. Gilson et al [22] performed a field study with Australian truck drivers, but they also did not have a control group. Pirolli et al [27] designed an experiment with 2×2 conditions but also worked without a control group. The remaining 7 studies did not use either an RCT or a field study. Specifically, He et al [14] collected secondary data from 15,310 WeChat group users to study weight loss, and used the propensity score method. Rodrigues et al [29] and Lieffers et al [25] used survey methods, whereas Bejar et al conducted a cross-sectional survey spanning 28 days [40]. Mummah et al [26] used a qualitative interview method, whereas Rollo et al [38] observed 10 patients with type 2 diabetes mellitus. Smith et al [39] studied beverage intake behaviors of 110 young adults over a 3-day period, and checked the volume of urine thereafter. In terms of duration, about 53% (16/30) of the studies covered a period of 3 months or more [11-14,19,20,22-24,28, 30-32,36,41,42], whereas 14 studies were conducted over less than 3 months [21,25-27,29,33-35,37-40,49,50].

### Characteristics of Inputs

Most of the studies (ie, 24) used only the text-based input mode [11-13,19-22,24-36,39-41,42]. For example, users logged their dietary behaviors by entering text about what they ate on a mobile app during the study period. On the contrary, 6 studies used more advanced input modes, including photo recognition [23,37,38,42,49], voice logging [37], and social network service message logging [14]. Although all the studies in our review acquired data on diet intake, some studies also collected other types of data, such as weight [11-14,19,26,31-34,37,41,50]. Studies also captured other health measures such as body mass index (BMI; by using weight and height) [11,12,34,37] and waist circumference [12], as well as medication [36]. Moreover, 6 of the studies [11,19,27,34,49,50] included goal settings in their inputs.

### Characteristics of Interventions

#### Mode and Theory of Mobile Interventions

On the basis of input data, the studies in our review provided the various modes of interventions as shown in Multimedia Appendix 1. We found that 12 studies [19,22,23,26,27,30,35-40] showed only the logged history of the user, which we refer to as *in-app log* mode. On the contrary, 18 studies [11-14,20,21,24,25,28,29,31-34,41,42,49,50] also included the feedback function in addition to the logged history, coded as “in-app feedback.” For example, the mobile app could send in-app feedback messages to users either as a tailored feedback on their progress or as a notification to keep users updated. Among these 18 studies, 5 studies used multiple modes of interventions. Specifically, 1 study used a push notification from the app [33] which was coded as “notification from mobile app” and 4 studies used other forms which was coded as “other notification,” that is, short message service notification [12,24,31], telephone calls [31], emails [11,12,31], Facebook messages [12].

Furthermore, some interventions in the included studies applied various theories for the design of their behavior change techniques. Specifically, 11 studies [11-13,19,21,23,24,26,31-33] included interventions based on theories, including learning theory (LT), theory of planned behavior (TPB), social cognitive theory (SCT), self-regulation theory (SRT), theory of behavior change (TBC), control theory (CT), self-determination theory (SDT), social network theory (SNT), and the processes-of-change theory (PCT). The most frequently used theory was the SCT (used in 5 studies) [11,12,19,26,32], followed by LT [11,13,26], SRT [11,19,26], and TPB [11,26,33], which were used in 3 studies each. In addition, 2 studies each used TBC [11,21], CT [12,23], and SDT [23,24], whereas 1 study each was based on PCT [31] and SNT [12].

#### Content of Mobile Interventions

The contents of the interventions in our review studies were categorized into 6 types (ie, self-monitoring, feedback, gamification, goal reviews, social support, and educational information), as seen in Table 1 and Multimedia Appendix 1. In Table 1, they were further divided into studies evaluating the effects of the interventions on (1) only dietary behavior changes, (2) only health outcomes, and (3) both.

In the *self-monitoring* category, all 30 studies contained diet-logging functions as part of their mobile interventions. Further, 12 studies [11-13,20,21,25,28,29,31,33,41,50] provided more detailed results including progress, of which 4 studies [20,21,28,29] assessed their effects on dietary behavior change, 3 studies [13,25,31] on both dietary behavior change and health outcomes, and the remaining 5 studies on health outcomes only. Interestingly, 2 studies [12,13] also provided functions for improving users’ adherence to healthier diets and recommended calorie prescriptions. Furthermore, 1 study used an intervention of sending messages about the status of users’ weight to increase user engagement [12].

*Feedback-based* interventions were seen in 18 studies [11-14,20,21,24,25,28,29,31-34,41,42,49,50]. Feedback is differentiated from self-monitoring in terms of its scale of interactions. Feedback intends to change users’ beliefs by providing a high level of interaction [53]. With respect to their effects, 6 studies [20,21,24,28,29,49] assessed the impacts of these interventions on dietary behavior change, 4 studies [13,25,31,32] on both dietary behavior change and health outcomes, and the remaining 8 studies on health outcomes only. As for intervention content, all 18 studies provided both progress reviews and recommendations, whereas 4 studies provided reminders [27,30,33,39].

In the *gamification* category, we identified and coded for 7 key elements of gamification: points, leaderboard, levels, quests and challenges, progression, viral loop [54], and trading [55].

We found 10 studies [11,14,19,23,27,31,32,34,49,50] that include gamification elements. Among them, 9 used progression elements [11,14,19,27,31,32,34,49,50], whereas 2 studies also provided quest and challenge elements [14,50]. Furthermore, 2 studies provided a leaderboard in their interventions, on the basis of points given to users [23,32]. There were no studies with gamification elements of level, viral loop, and trading in our review. With respect to their effects, 4 studies [19,23,27,49] assessed the impacts of these interventions on dietary behavior change, 3 studies [11,31,32] on both dietary behavior change and health outcomes, and the remaining 3 studies on health outcomes only.

In the category of *goal review*, 5 studies [11,19,27,34,50] with goal setting inputs provided a review of goals. Among these, 1 study [11] supported users by providing goal progress reports. In sum, 2 studies [19,27] assessed intervention impacts on dietary behavior change, whereas the remaining 3 studies evaluated effects on health outcomes.

In the *social support* category, 3 studies [14,23,32] used interventions that provide such support. Compared with self-monitoring, feedback, and gamification categories, the goal review and social support categories of interventions were less used. With respect to their effect, 1 study [23] assessed the impact of social support on dietary behavior change, another study [32] on both dietary behavior change and health outcomes, and the remaining study on health outcomes only, that is, weight loss. In terms of content, all 3 studies provided general social support, whereas 1 study [23] provided comparison functions among peers in its intervention.

The last category of mobile interventions consisted of those providing *educational information*. Here, 2 studies [19,35] provided educational materials on diets and the challenges to adhere to the prescribed diets. Furthermore, 1 study [19] assessed the effect of the interventions on dietary behavior change, whereas the other study examined health outcomes only.

**Table 1:** Content of interventions and effects.

Content of interventions		Studies with interventions on dietary behavior changes only (N=12), n (%)	Studies with interventions on both dietary behavior changes and health outcomes (N=5), n (%)	Studies with interventions on health outcomes only (N=13), n (%)
**Self-monitoring**		12 (100)	5 (100)	13 (100)
	Logging	12 (100)	5 (100)	13 (100)
	Progress	4 (33)	3 (60)	5 (38)
	Adherence	0 (0)	1 (20)	1 (8)
	Engagement	0 (0)	0 (0)	1 (8)
**Feedback**		6 (50)	4 (80)	8 (62)
	Reviews on their progress	6 (50)	4 (80)	8 (62)
	Recommendations	6 (50)	4 (80)	8 (62)
	Reminders	2 (17)	0 (0)	2 (15)
**Gamification**		4 (33)	3 (60)	3 (23)
	Progression	3 (25)	3 (60)	3 (23)
	Quest and challenge	0 (0)	0 (0)	2 (15)
	Leaderboard	1 (8)	1 (20)	0 (0)
	Points	1 (8)	1 (20)	0 (0)
**Goal reviews**		2 (16)	0 (0)	3 (23)
	Reviews of goal logs	2 (16)	0 (0)	3 (23)
	Goal progress support	0 (0)	0 (0)	1 (8)
**Social support**		1 (8)	1 (20)	1 (8)
	Social support	1 (8)	1 (20)	1 (8)
	Social comparisons	1 (8)	0 (0)	0 (0)
Educational information		1 (8)	0 (0)	1 (8)

### Characteristics of Outcomes

The mode of measurement of outcomes in the included studies (24 studies) was mostly self-reported by participants themselves [12-14,19-22,24-34,36,37,40,41,49,50]. Among the 30 studies, 2 studies measured the outcome variable by a blood test [11,42] and 2 studies measured it by a urine volume or sodium test [35,39]. Furthermore, 2 studies measured the participants’ weight by a scale in a lab [11,38], and 2 studies provided a photo recognition mode for users to report the outcome [23,49].

Regarding the outcomes assessed by the 30 papers, 12 studies [19-24,27-30,40,49] evaluated dietary behavior change as their only outcome. On the contrary, 13 studies [11,12,14,33-39,41,42,50] directly examined the effects of mobile interventions on health outcomes, whereas 5 studies [13,25,26,31,32] assessed the effects on both dietary behavior change and health outcomes.

#### Dietary Behavior Change

As mentioned above, 12 studies aimed to change users’ dietary behavior as their main outcome. Indeed, we found that the dietary behaviors examined in the studies were quite diverse. Several studies focused on the intake of specific food types, including high-fiber bread and low-fat milk [19], vegetables [27], fats [23], as well as low-calorie foods [29,49]. However, most of the studies [20,21,22,24,28,30,40] focused on healthy food intake defined by different combinations of fruit, vegetable, processed food, sugar, fat, salt, sugar-sweetened drinks, calories, and, lastly, a Mediterranean diet.

#### Health Outcomes

On the contrary, 13 studies [11,12,14,33-39,41,42,50] assessed the direct effect of mobile interventions on users’ health outcomes, that is, health status changes and biochemical outcomes. Of these, 9 studies [11,12,14,33,34,37,38,41,50] measured health status outcomes in terms of weight loss and BMI. Furthermore, 6 studies measured biochemical outcomes, including blood glucose level [11,36,42], urine volume [39], urinary sodium [35], and blood pressure/hemoglobin [34].

#### Dietary Behavior Change and Health Outcomes

In our review, 5 studies [13,25,26,31,32] aimed at dietary behavior change as well as achieving better health outcomes. These studies are rare, but important, because they show the relationship between dietary behavior changes and health outcomes. For this reason, we investigated each study in detail. First, *Martin et al* [13] examined the effect of a weight loss intervention that delivers personalized recommendations and educational materials via the multimedia capabilities of participants’ smartphones. They found that the participants successfully adhered to their calorie intake prescriptions provided by the intervention, which resulted in weight loss at the end of 12 weeks. *Lieffers et al* [25] examined the effects of self-monitoring by using logs of food and calorie intakes, as well as recipes, exercise, and restaurant nutrition information. They surveyed dietitians to evaluate the effectiveness of the recommendations on nutrition and food apps. They reported that 41% of dietitians felt the studied apps would help users in managing their body weight and result in healthier body composition. *Mummah et al* [26] found that mobile-based self-monitoring resulted in changes in users’ vegetable intake behavior, thereby achieving weight loss. The RCT tested the effect of theory-driven mobile interventions using 18 behavior change techniques. *Hales et al* [32] reported that social support using a social network function resulted in users’ dietary behavior change, that is, consuming fewer calories, and health outcomes, that is, weight loss. *Hebden et al* [31] developed a custom program to provide personalized coaching as their intervention. Their study found that the intervention changes the types of food consumed, thereby improving health outcomes, such as weight loss.

## Discussion

Our scoping review aimed at identifying and synthesizing prior studies of mobile-based interventions for dietary behavior change and health outcomes. The implications of the findings of this review are discussed below along with the strengths and limitations.

### Principal Findings

From our review, we identified the most common input mode as text-based input. However, using more advanced methods, such as photo recognition, can ease the burden of diet input and logging. Furthermore, although all the reviewed studies captured users’ diet intake, future apps could benefit from collecting other types of data, including demographics (eg, height and weight), medication (eg, insulin dosage), health measures (eg, BMI), and goal setting.

Moreover, we found that self-monitoring, followed by personalized feedback type of mobile intervention, was most common. Although both self-monitoring and personalized feedback were found to help in achieving the desired dietary behavior changes, the other (less common) content categories of gamification, goal reviews, social support, and educational information were also helpful in this regard. Thus, mobile apps in future can make better use of these other intervention categories.

In our review, all the 11 studies [11-13,19,21,23,24,26,31-33] stating that their interventions were based on behavior change theories made little explicit reference to theory. Of these, 10 studies merely mentioned the theories but did not describe how the theoretical constructs were used to derive their interventions. As an exception, only 1 study [26] explained how behavioral theories were used to derive their intervention approach, but did not examine the underlying mechanisms of the behavior change. Moreover, the remaining 19 studies in our review made no reference to theory. This suggests the need for more theory-based interventions for dietary behavior change. This is because theoretical models provide links between intervention content and mediating processes implied by theory. They can enable the identification of features that systematically influence the effectiveness of interventions and, hence, help build a cumulative understanding of what works and how [56]. Without understanding the underlying mechanisms of behavior change techniques, decision makers lack information to make choices about what interventions are likely to be effective in their own settings.

The studies we reviewed investigated how their mobile interventions affected dietary behavior changes and/or health outcomes. However, there was little consistency among the dietary behavior changes examined, suggesting that more comprehensive and consistent measures can be developed for this purpose. Furthermore, in terms of health outcomes, most studies have focused on assessing weight loss. Although weight loss is an important measure of health improvement, other outcomes may also need to be examined.

We also found that most of the studies did not focus on dietary behavior change as a mediator for health outcomes. Although weight loss or blood glucose control is crucial for some user groups, such as obese people or diabetics, a healthy diet helps to improve the overall health for most people. Thus, we need to understand the mechanisms behind mobile-based interventions’ effects on dietary behavior changes and health outcomes. This limitation of existing literature is somewhat related to the current underutilization of theory-based mobile interventions.

### Strengths and Limitations

One of the strengths of this research is that by following the objective of scoping reviews, this study synthesizes the extant literature and highlights potential gaps in it. We provide a comprehensive map of the literature on the underexplored topic of mobile interventions for dietary behavior change. The review covers aspects related to study characteristics, input mode and contents, mode and content of mobile interventions, related behavioral theories, and outcomes. A number of gaps in this area are identified in the Discussion section above based on our review.

Compared with prior scoping reviews in this domain, another strength of our review is that it identifies the underinvestigated mediating process of dietary behavior change in the relationship between mobile interventions and health outcomes. Furthermore, previous reviews have not incorporated a wide range of studies concerning the effect of mobile interventions on dietary behavior change, as we do. Specifically, they did not provide a descriptive overview by synthesizing studies [15,43], did not include a broad range of study designs and methodologies [43], or focused on a limited scope, that is, vegetable intake [16]. Thus, our review adds to the literature by providing a more comprehensive view of mobile-based interventions for dietary behavior changes and their outcomes.

One limitation of this scoping review is that potential biases might have influenced the results. *First,* publication bias could be present, indicated by the absence of negative effects of reported interventions included in this review. There was only 1 ineffective study [38], which failed to show the weight loss of users from using the mobile app. Although measuring the effectiveness of the interventions is not within the scope of our study, we mainly focused on identifying the relationship between mobile interventions, dietary behavior changes, and health outcomes. *Second,* we observed that the interventions described in earlier studies [29,30,31,33,36,41] in 2013, differ from the interventions described in newer studies, [14,27,35,37] in 2013. Compared with the recent apps with advanced technologies such as self-tracking sensors, food photo recognition, and customized real-time feedback, the apps with older interventions might not prove to be as effective. *Finally,* the search criteria we used for retrieving the studies were very broad and initially started with a large number (5607) of studies. As there is no consistent terminology for dietary behavior changes and outcomes, we tried to cover all the aspects that have been studied in this regard.

### Conclusions

To the best of our knowledge, our scoping review provides the first overview of the relationships among mobile-based interventions, dietary behavior change, and health outcomes. In contrast to the general belief of the importance of dietary behavior changes, not many studies have examined dietary behavior changes as a mediator for health outcomes. Future research needs to be conducted to understand the effects of mobile interventions for dietary behavior changes on health outcomes.

## Appendix

Multimedia Appendix 1 Summary of included studies.

Multimedia Appendix 2 Supplementary information of hand-search process.

## Abbreviations

BO: biochemical outcome
BMI: body mass index
CT: control theory
DBC: dietary behavior change
HS: health status
JMIR: *Journal of Medical Internet Research*
LT: learning theory
mHealth: mobile health
PCT: processes-of-change theory
RCT: randomized controlled trial
TBC: theory of behavior change
TPB: theory of planned behavior
SCT: social cognitive theory
SDT: self-determination theory
SNT: social network theory
SRT: self-regulation theory
