# Supplementary material for: Mobile-Based Interventions for Dietary Behavior Change and Health Outcomes: Scoping Review
Source: JMIR Mhealth Uhealth. 2019 Jan 21;7(1):e11312. doi: 10.2196/11312 (PMC6360385; doi:10.2196/11312)
Supplement: Multimedia Appendix 1 [file mhealth_v7i1e11312_app1.pdf]

Multimedia Appendix 1

Summary of included studies

First Author and Year	Study Characteristics		Input		Intervention			Outcome	
	Participants (Sample Size, Mean Age)	Study Method, Duration	Mode	Content	Mode	Content	Theory **	Mode of measurement	Content*
Block et al., 2015 [11]	U.S. Pre-diabetic patients (N=339, Age=55)	RCT, 1 Year	Text	Weekly weight goal, Diet, Weight, Height	In-App log, In-App feedback, Emails (other notification)	Personalized feedback on diet and risk of diabetes, Goal reviews, Gamification (Progression)	LT, TPB, SCT, SRT, TBC	Blood test and weight check by a scale in a lab	**HS:** Weight, BMI **BO:** Glucose level
Patrick et al., 2014 [12]	U.S. Obese college students (N=22, Age=22)	RCT, 6 Months	Text	Waist circumference, Diet, Weight, Height	In-App log, In-App feedback, Facebook, SMS, Emails (other notification)	Personalized feedback on diet and weight	SCT, CT, SNT	Self-reported	**HS:** Weight
Martin et al., 2015 [13]	U.S. Obese adults (N = 40, M = 44)	RCT, 12 Weeks	Text	Diet, Weight	In-App log, In-App feedback: "SmartLoss"	Personalized feedback on diet	LT	Self-reported	**HS:** Weight **DBC:** Dietary adherence (The changes of the number of days in the calorie zone of adherence, Zone: by fitting an upper and lower curve through the mean absolute error from the recommended calories for women: 1200 kcal/d, and for men: 1400 kcal/d
He et al., 2017 [14]	China, Adults in a WeChat group for weight loss (N=15,310, Age=35)	Propensity score methods, 6 Months	Text message in WeChat group	Diet, Weight	In-App log, In-App feedback in WeChat Group (SNS)	Personalized feedback on weight, and diet, Social support, Gamification (Progression , Quest and challenges)	-	Self-reported	**HS:** Weight
Duncan et al., 2014 [19]	Australia, Middle-aged men (N=301, Age=44)	RCT, 9 Months	Text	Diet, Goals of diet, Weight	In-App log: "'ManUp"	Educational information on diet, Goal reviews, Gamification (Progression)	SCT, SRT	Self-reported	**DBC:** Healthy food intake (the changes in the proportion of participants consuming higher-fiber bread and low-fat milk per day)

Elbert et al., 2016 [20]	Netherlands, Adult (N=146, Age=41)	RCT, 6 Months	Text	Diet (Fruit and Vegetable)	In-App log, In-App feedback, Audio/ Text messages	Personalized feedback of diet	-	Self-reported	**DBC:** Fruit and vegetable intake (the change in the amount of consuming fruit and vegetable per week)
Gilliland et al., 2015 [21]	Canada, Adult (N=208, Age=33)	RCT, 10 Weeks	Text	Diet	In-App log In-App feedback	Personalized feedback of diet	TBC	Self-reported	**DBC:** Healthy food intake (the changes in the amount of consuming seasonal/ local foods, vegetables, fruits, processed foods, sugar, fat, and salt per day)
Gilson et al., 2016 [22]	Australia, Truck drivers (N=44, Age=48)	Field study, 20 Weeks	Text	Diet	In-App log	Only review logs, Comparison with other users	-	Self-reported	**DBC:** Healthy food choices (the changes in the amount of consuming saturated fat and sugar per week)
Helander et al., 2014 [23]	Finland, Active users (N=4,895, Age= -)	RCT, 6 Months	Photo recognition and text	Diet	In-App log, free iPhone app "The Eatery"	Only review logs, Peer feedback of diet (Social support), Gamification (Points, Leaderboard)	CT, SDT	Photo recognition	**DBC:** Healthy food intake (the changes in the amount of consuming fat per day)
Kerr et al., 2016 [24]	Australia, Young adults: university students (N=247, Age=21)	RCT, 6 Months	Text	Diet (Fruit and vegetables, sugar-sweetened beverage)	In-App log, In-App feedback, weekly text messages	Personalized feedback on diet	SDT	Self-reported	**DBC:** Healthy food intake (the changes in the amount of consuming vegetables, fruit, sugar-sweetened beverages per day)
Lieffers et al., 2014 [25]	Canada, Dietetics' app users (N=139, Age=32)	Survey, One time	Text	Dietetic practice in mobile devices/apps	In-App log, In-App feedback	Personalized feedback on nutrients consumed	-	Self-reported	**HS:** Varied status changes **DBC:** Varied behavior changes (food with fewer calories, a proper amount based on exact information)
Mummah et al., 2016 [26]	U.S. Overweight adults (N=18, Age=43)	Qualitative Interview	Text	Diet (Vegetable), Weight	In-App log	Only review logs of diet	LT, TPB, SCT, SRT	Self-reported	**HS:** Weight **DBC:** Vegetable intake (the

									changes in quantity (i.e., servings) and variety (i.e., types) of consuming vegetables per a serving)
Pirolli et al., 2017 [27]	U.S. Mobile users recruited at Univ., Craigslist, and Nextdoor. (N=64, Age=30)	Field study, 28 Days	Text	Diet, Goals of diet	In-App log: "PARC Coach"	Only review logs of diet, Reminders, Goal reviews, Gamification (Progression)	-	Self-reported	**DBC:** Vegetable choices (the changes in the amount of consuming vegetables per day)
Recio-Rodriguez et al., 2016 [28]	Spain, Adults in primary care centers (N=833, Age=51)	RCT, 3 months	Text	Diet	In-App log, In-App feedback	Personalized feedback on diets	-	Self-reported	**DBC:** Mediterranean food intake (the changes in the average amount of consuming Mediterranean diet per day)
Rodrigues et al., 2013 [29]	Portugal, Adults who use application "SapoFit" (N=100, Age=-)	Survey, One time	Text	Diet	In-App log: "SapoFit", In-App feedback	Personalized feedback on diets	-	Self-reported	**DBC:** Healthy food intake (the changes in the amount of consuming calories before and after a survey)
Turner-McGrievy et al., 2013 [30]	U.S. Obese adults (N=96, Age=44)	RCT, 12 months	Text	Diet	In-App log: "MOVE!"	Only review logs of diet, General podcasts reminders	-	Self-reported	**DBC:** Healthy food intake (the changes in the mount of consuming calories, fat, added sugar, fruit, vegetables per day and week)
Hebden et al., 2013 [31]	Australia, Young adults obese patients (No = 51, Age = 27)	RCT, 36 Weeks	Text	Diet (Fruit, vegetables, beverages, and total energy), Weight	In-App log, In-App feedback, SMS notifications, calls, emails (other notification)	Personalized feedback on diet, Motivational advice, Gamification (Progression)	PCT	Self-reported	**HS:** Weight **DBC:** Healthy food intake (the changes in the frequency of consuming specific foods and drinks i.e., fruit, vegetables, energy-dense take-away meals, SSB, and water, per day)
Hales et al., 2016 [32]	U.S. Overweight adults in a weight loss	RCT, 12 Weeks	Text	Diet, Weight	In-App log In-App feedback,	Personalized feedback of diet, Social	SCT	Self-reported	**HS:** Weight **DBC:** Healthy food intake (the

	program (No=51, Age=48)				(SNS): "Social POD"	support, Gamification (Progression, Points, Leaderboard)			changes in the average amount of consuming calories per day and week)
Brindal et al., 2013 [33]	Austria, Obese women (N=58, Age=42)	RCT, 8 Weeks	Text	Diet, Weight	In-App log, Push notification (notification in mobile app)	Personalized feedback, and Reminders of Diet, Weight	TPB	Self-reported	**HS:** Weight
Goh et al., 2015 [34]	Singapore, Patients with type 2 diabetes mellitus (N=84, Age=48)	RCT, 8 Weeks	Text	Diet, Goals of diet, Weight, Height	In-App log and In-App feedback, "iDAT"	Personalized feedback of diet, Goal reviews, Gamification (Progression)	-	Self-reported	**HS:** BMI<br>**BO:** Blood pressure, and hemoglobin
Ipjian et al., 2017 [35]	U.S. Free mobile app active users (N=15, Age=35)	RCT, 4 Weeks	Text	Diet	In-App log, "MyFitnessPal"	Educational information on reducing sodium intake	-	Urine sodium test in a lab	**BO:** Urinary sodium
Kirwan et al., 2013 [36]	Australia, Patients with type 1 diabetes (N=72, Age=35)	RCT, 6 Months	Text	Diet, Blood glucose, Insulin dosage, Medication	In-App log	Review logs of diet, Blood glucose, Insulin dosage	-	Self-reported	**BO:** Blood glucose
Pendergast et al., 2017 [37]	Australia, Young adults recruited online or offline (N=90, Age=25)	RCT, 4 Days	Photo recognition, voice and text	Diet (Food and beverage), Weight, Height	In-App log: "FoodNow."	Only review logs of diets	-	Self-reported	**HS:** BMI
Rollo et al., 2015 [38]	Australia, Patients with type 2 diabetes mellitus (N=10, Age=54)	Experiment, 12 days	Photo recognition	Diet	In-App log "NuDAM"	Only review logs of diets	-	Weight check by a scale in a lab	**HS:** Weight
Smith et al., 2014 [39]	China, Young adults (N=110, Age=30)	Experiment, 3 days	Text	Diet (Food and beverage)	In-App log	Only review logs of diets and general 24-hour reminders	-	Urine volume test in a lab	**BO:** Urine volume
Bejar et al. 2016 [40]	Spain, University students (N=119, Age = 22)	Protocol, 28 days	Text	Diet (Drink and Food)	In-App log	Only review logs	-	Self-reported	**DBC:** Healthy food intake (the changes in the average amount of consuming a sugary drink and a variety of food products per day)
Spring et al., 2013 [41]	U.S. Obese adults at a hospital (N=69, Age=58)	RCT, 12 months	Text	Diet, Weight	In-App log, In-App feedback	Personalized feedback on diets	-	Self-reported	**HS:** Weight
Waki et al., 2015 [42]	Japan, Patients with Type 2 Diabetes using "DialBetics" (N=54, Age=59)	RCT, 3 months	Photo recognition and Text	Diet	In-App log: "DialBetics", In-App feedback	Personalized feedback on diets	-	Blood screening in a lab	**BO:** Blood glucose

Wharton et al., 2014 [43]	U.S. Adults in an 8-week weight loss trial (N=19, Age=42)	Field study, 8 weeks	Text	Diet, Goals of diet and weight	In-App log: "Loose it!" , In-App feedback	Personalized feedback on diets, Goal reviews, Gamification (Progression, Quest and challenges)	-	Self-reported	**HS:** Weight
Rabbi et al., 2015 [45]	U.S. Mobile users recruited at Univ. (N=17, Age=28)	RCT, 3 weeks	Text, Photo recognition	Diet, Goals of diet	In-App log: "MyBehavior", In-App feedback	Personalized and automated feedback on diet, Gamification (Progression)	-	Self-reported, photo recognition	**DBC:** Healthy food intake (the changes in the average amount of consuming calories per day)

* Dietary behavior changes (DBC), Biochemical outcomes (BO), Health status (HS)

** Learning Theory (LT), Theory of Planned Behavior (TPB), Social Cognitive Theory (SCT), Self-Regulation Theory (SRT), Theory of Behavior Changes (TBC), Control Theory (CT), Self-Determination Theory (SDT), Social Network Theory (SNT), Processes-of-Change Theory (PCT)
