# Supplementary material for: Mobile-Based Interventions for Dietary Behavior Change and Health Outcomes: Scoping Review
Source: JMIR Mhealth Uhealth. 2019 Jan 21;7(1):e11312. doi: 10.2196/11312 (PMC6360385; doi:10.2196/11312)

Multimedia Appendix 2

Supplementary information of hand-search process

Figure A1. Flowchart of hand-search process for Theme 188

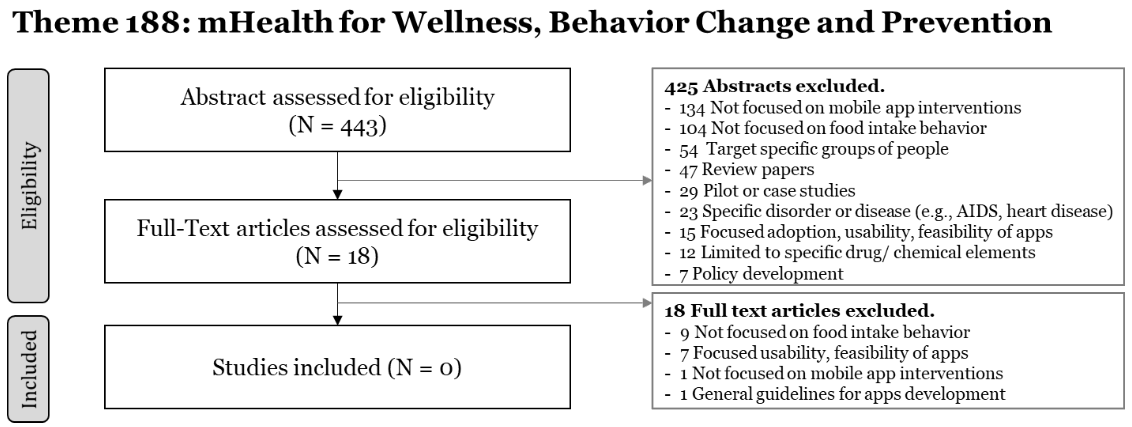

Figure A2. Flowchart of hand-search process for Theme 457

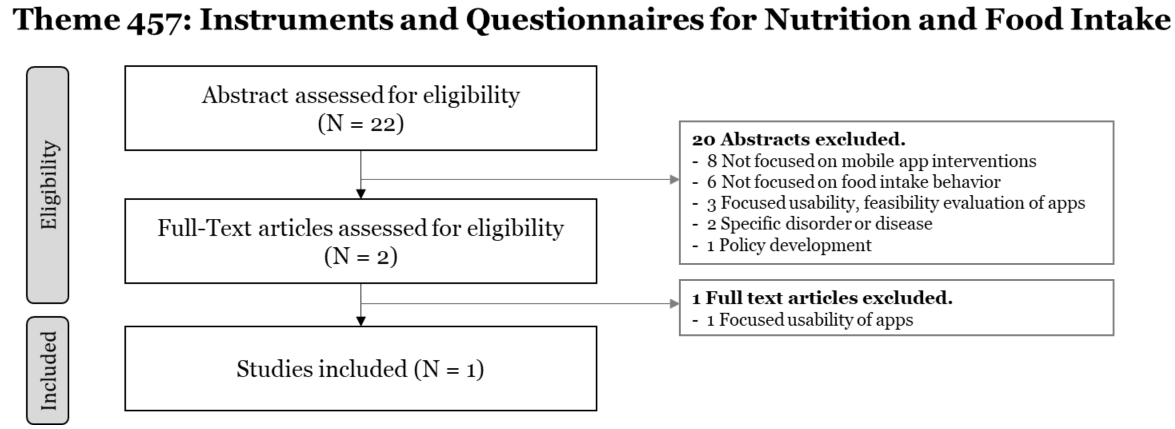

Figure A3. Flowchart of hand-search process for Theme 51

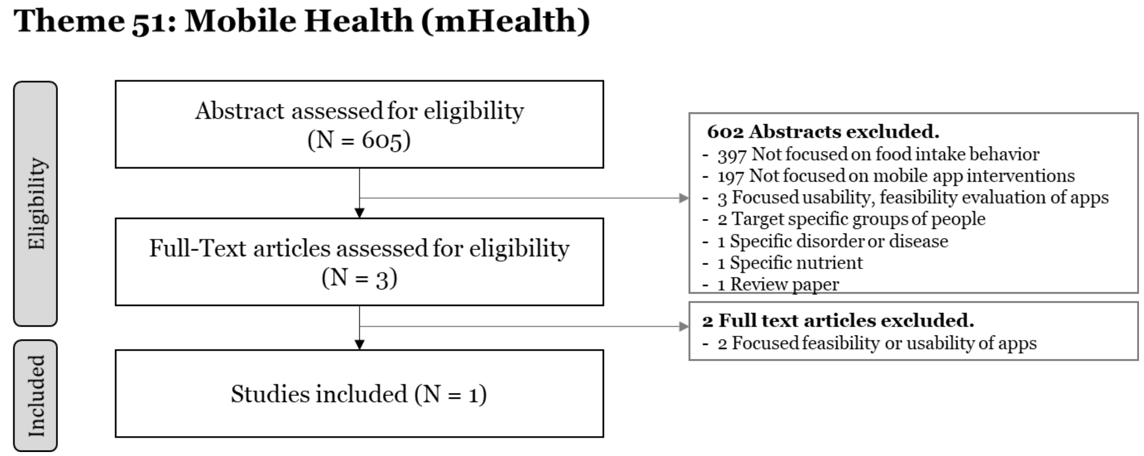

Supplement: Multimedia Appendix 2 [file mhealth_v7i1e11312_app2.pdf]
